# Immunization of broiler chickens with five newly identified surface-exposed proteins unique to *Clostridium perfringens* causing necrotic enteritis

**DOI:** 10.1038/s41598-023-32541-4

**Published:** 2023-03-31

**Authors:** Sara Heidarpanah, Alexandre Thibodeau, Valeria R. Parreira, Sylvain Quessy, Mariela Segura, Ilhem Meniaï, Marcelo Gottschalk, Annie Gaudreau, Tristan Juette, Marie-Lou Gaucher

**Affiliations:** 1 Chaire de Recherche en Salubrité des Viandes (CRSV), Département de Pathologie et Microbiologie, Faculté de Médecine Vétérinaire, Université de Montréal, Montréal, Canada; 2 Groupe de Recherche Sur les Maladies Infectieuses en Production Animale (GREMIP), Faculté de Médecine Vétérinaire, Université de Montréal, Montréal, Canada; 3 Swine and Poultry Infectious Diseases Research Centre (CRIPA), Faculté de Médecine Vétérinaire, Université de Montréal, Montréal, Canada; 4 Food Science Department, Canadian Research Institute for Food Safety (CRIFS), University of Guelph, Guelph, ON N1G 2W1 Canada; 5 Faculté de Médecine Vétérinaire, Université de Montréal, Montréal, Canada

**Keywords:** Microbiology, Vaccines

## Abstract

Since the ban or reduction on the use of antibiotic growth promoters (AGPs) in commercial broiler chickens in many countries, avian necrotic enteritis (NE) caused by *Clostridium perfringens* has re-emerged as one of the biggest threats for the poultry industry worldwide. While the toolbox for controlling NE in the absence of antibiotics consists of a limited number of alternatives for which the overall effectiveness has yet proven to be suboptimal, an effective vaccine would represent the best control strategy for this often-deadly disease. Using a comparative and subtractive reverse vaccinology approach, we previously identified 14 putative antigenic proteins unique to NE-causing strains of *C. perfringens*. In the current work, the in silico findings were confirmed by PCR and sequencing, and five vaccine candidate proteins were produced and purified subsequently. Among them, two candidates were hypothetical proteins, two candidates were prepilin proteins which are predicted to form the subunits of a pilus structure, and one candidate was a non-heme iron protein. Western blotting and ELISA results showed that immunization of broiler chickens with five of these proteins raised antibodies which can specifically recognize both the recombinant and native forms of the protein in pathogenic *C*. *perfringens*.

## Introduction

Avian necrotic enteritis (NE) is a complex and multifactorial enteric disease of commercial chickens and turkeys that has major economic consequences on the poultry industry worldwide^1,2^. The primary cause is pathogenic strains of type G *Clostridium perfringens*^3^, a Gram-positive, anaerobic, and spore-forming bacterium that is widely distributed in various environments, such as soils, feces, foods, and intestine of both humans and animals ^4^. In broiler chickens, the sole presence of pathogenic strains of *C. perfringens* has been shown to be insufficient to reproduce NE in chicken disease models, and it is recognized that different predisposing factors including *Eimeria* infection, high amounts of non-starch polysaccharides, immunosuppression, and high amounts of animal proteins (e.g., fishmeal) in the diet are required for the onset of NE^5^.

To date, various *C. perfringens*-associated toxins and other virulence factors have been identified as contributors to NE development, and a large proportion of them have also been evaluated for their ability to stimulate a protective immunity in the avian bird^6,7^. Although some of these tested antigens have proven to stimulate a protective immune response, none of them has proven to be fully protective against the disease in broiler chickens^8,9^.

When breaking NE pathogenesis down, the first step in the establishment of the intestinal infection by *C. perfringens* is the displacement of commensal *C. perfringens* by bacteriocins, with perfrin having been identified to play an important role^10,11^. The subsequent phase would involve the attachment of disease-causing *C. perfringens* to the intestinal mucosa through the expression of different adhesion factors, including pili (e.g., FimA and FimB), collagen adhesion proteins (e.g., CnaA) and fibronectin-binding proteins (e.g., FbpA and FbpB) for which the role has recently been studied^12,13^. Over the past few years, colonization followed by degradation of the mucus layer covering the small intestine of the chicken host by *C. perfringens* extracellular enzymes including zinc metalloproteases, collagenolytic enzymes, glycoside hydrolases, sialidases, and other degradative enzymes became a major key step of interest for scientists^10^. As part of better understanding NE pathogenesis, the identification of genetic determinants of *C. perfringens* that could play a role in both the ability of the pathogen to cause the disease and to elicit a protective immune response has been established as a priority^14,15^. Indeed, when adopting a global approach aiming at reducing the impacts of NE in the absence of antibiotic growth promoters (AGPs), vaccination represents the most cost-effective alternative^16^.

As of today, most efforts have focused on designing vaccines based on alpha and NetB toxins. The only commercially available vaccine (Netvax®) which was based on alpha-toxin (CPA) toxoid and administered to broiler breeder hens subcutaneously, is no longer authorized^17,18^. On the experimental side, the efficacy of the recombinant form of NetB (rNetB) as a single subunit vaccine candidate, the formalin-treated bacterin and the toxoid to protect chickens from NE was also investigated. Findings indicated that immunization with all of these components was not able to protect birds against a severe challenge, nor the immunization scheme used was applicable to field conditions^8^. Immunization with *Salmonella*-vectored vaccines delivering nontoxic fragments of the alpha toxin, of NetB, of a fructose 1,6-bisphosphate aldolase (FBA)^19^, of a pyruvate-ferredoxin oxidoreductase (PFOR), and of a hypothetical protein (HP) provided partial protection against a challenge with virulent *C. perfringens* and required the improvement of antigen expression systems in *Salmonella* for effective delivery of antigens^20^. Other subunit vaccines involving TpeL, an endo-beta-N-acetylglucosaminidase (Naglu), a phosphoglyceromutase (Pgm)^21^, and a glyceraldehyde 3-phosphate dehydrogenase (GPD) proteins^22^, and three predicted pilin structural subunits (CnaA, FimA, FimB) of *C. perfringens* have also been evaluated, with suboptimal results regarding the ability of these antigens to stimulate an immune response providing complete protection against NE in broiler chickens^23^.

While all the research work conducted on NE vaccine development so far relied on classical vaccinology, the ease of access to high-throughput sequencing technologies generating significant amounts of useful information within a very short period of time now gives way to innovative alternatives, including among others, comparative and subtractive reverse vaccinology (CSRV)^24^. CSRV was introduced more than twenty years ago and is one of the most cost-effective methods, not only to describe the proteome of a bacterial species, but also to allow for the rapid in silico identification of putative antigens unique to the disease-causing sub-population of this species^25^. The particular advantage of this approach is that depending on what needs to be documented, bioinformatics tools can be used alone or in combination to document specific characteristics of the bacterial proteome. For example, the nature, the localization, and the function of the proteins forming the bacterial proteome are just a few^26^. The first bacterial vaccine developed with the use of reverse vaccinology to be successfully commercialized was the MenB-4C vaccine which targets *Neisseria meningitidis*, a Gram-negative bacterium that causes upper respiratory tract infections^27^. This vaccine consists of four surface-exposed antigens: Neisserial adhesin A (NadA), Neisserial Heparin Binding Antigen (NHBA), factor H binding protein (fHbp), and Outer Membrane Vesicles (OMV)^28^. By allowing for the rapid identification of surface-exposed proteins, structures that can be widely exposed to the immune system of the host^29^ and that can also play crucial roles in the pathogenesis of many diseases, especially during the colonization step^30,31^, CSRV appears as the most attractive way to rapidly identify protective candidate antigens in NE-causing *C. perfringens*. Using a CSRV approach on sixteen *C. perfringens* strains isolated from either healthy or NE-affected broiler chickens, our research group previously identified fourteen proteins unique to six NE-causing *C. perfringens* strains and absent from ten commensal *C. perfringens* strains from this same collection^32^. The objectives of the current study were then to (i) validate the results of this in silico study, (ii) produce recombinant proteins from the sequence encoding the selected candidate proteins identified, (iii) evaluate the ability of these proteins to initiate an immune response in immunized broiler chickens, and (iv) assess the ability of the generated antibodies to recognize both the selected candidate proteins in pathogenic *C*. *perfringens* and their recombinant form. In conclusion, this work was the first report utilizing a CSRV method to discover five surface-exposed vaccine targets unique to *C. perfringens* and subsequently evaluate their immunogenicity.

## Results

### Bioinformatics analysis

Bioinformatics analyses performed on P509 predicted a transmembrane helix between residues 12–34 and an N-terminal signal peptide which cleaved between positions 47 and 48. One transmembrane helix was also predicted between residues 13–35 in P561. The GRAVY score attributed to the full-length sequence of P509 and P561 was − 0.026 and − 0.33, respectively. When excluding transmembrane regions, these scores were modified to − 0.286 and − 0.732 for P509 and P561, respectively, which means that the truncated forms for P509 and P561 were approximately ten and two times more soluble than the full-length protein, respectively. Two transmembrane helices at positions 7–29 and 668–690 were found in the sequence of P2091, while the sequence of P1074 revealed one transmembrane helix at position 331–350. The GRAVY score of P1074 and P2091 were − 0.46 and − 0.44, respectively. On the other hand, no transmembrane helices and signal peptide were found in P153, P264, and P537. The gravy score attributed to P153, P264, and P537 were − 1.212, − 0.917, and − 0.816, respectively. The VaxiJen score for all proteins, except for P537 which was selected on the basis of its low immunogenic score as a negative control to be included in the in vivo immunization assay, was above 0.5. These candidate proteins were then considered as probable antigens. Vaxijen scores are presented in Table 1.

**Table 1:** Name, size, cell localization and VaxiJen score of candidate proteins that were successfully produced.

Name	Size (KDa)	Location	VaxiJen score
P153	5.6	Extracellular/cytoplasmic membrane	0.69
P264	9.2	Extracellular/cytoplasmic	1.47
P509	17.3	Extracellular	0.86
P537	21	Cytoplasmic	0.21
P561 (truncated form)	17	Extracellular	0.64

### Validation of in silico studies, amplification, and cloning of candidate protein-encoding genes

PCR results confirmed the presence of all 14 candidate protein-encoding genes, identified in our previous study, in each of the six virulent strains of *C. perfringens* screened. A BLAST alignment revealed 100% sequence similarity between 10 protein-encoding gene amplicons derived from virulent *C. perfringens* strains and in silico identified protein-encoding genes. PCR results also confirmed the existence of half of the candidate protein-encoding genes in commensal strains of *C. perfringens* (see Supplementary Table 1). The BLAST alignment showed 97%-99% sequence homology between these seven protein-encoding gene amplicons derived from commensal *C. perfringens* strains and in silico identified protein-encoding genes (see Supplementary Table 2). These candidate proteins were then excluded on the basis of the ubiquitous presence of *C. perfringens* in the gastrointestinal tract of broiler chickens and on the probability of this DNA similarity to lead to the expression of proteins showing identical functions (Supplementary Table 2). When taking PCR and sequencing results together, seven candidate proteins were kept for the next steps: P153, P264-2, P509, P537, P561, P1074, P2091. Among them, five candidate protein-encoding genes were successfully cloned into pET151/D-TOPO® vector. Several attempts for the cloning of P1074 and P509 genes in this vector were unsuccessful, and only the synthesis and cloning of P509 gene into pET-24a(+) vector was possible (GenScript, Piscataway, NJ, USA).

### Expression and purification of candidate proteins

**Figure 1:**
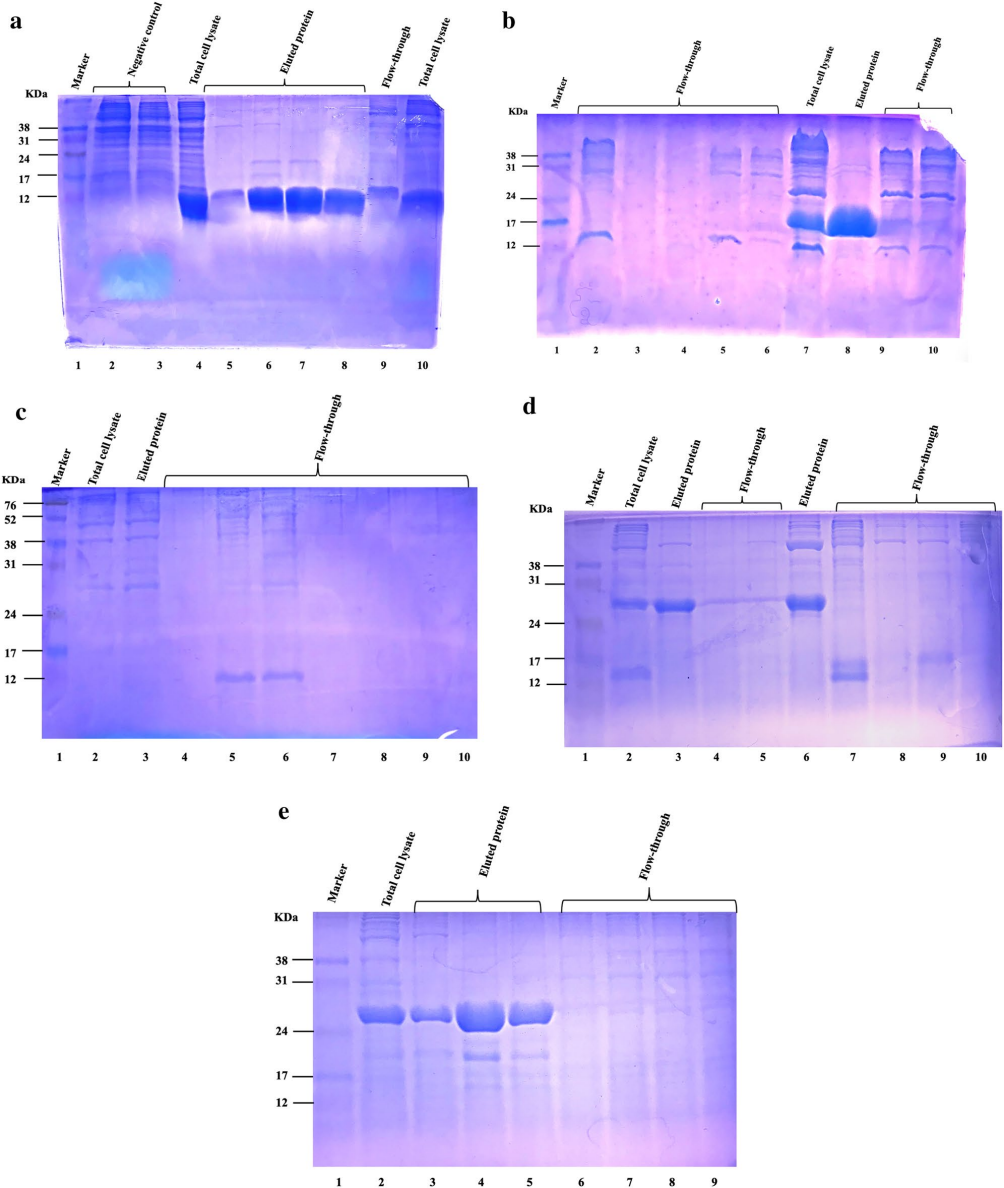
SDS-PAGE gel showing purification of His-tagged recombinant proteins by imidazole step gradient. 15 µL of total cell lysate and fractions of (**a**) P153, (**b**) P264, (**c**) P509, (**d**) P537, and (**e**) P561 were observed by SDS-PAGE and Coomassie Blue staining. The size of the recombinant protein is 4 KDa higher than the expected size due to the inclusion of the His-tag and V5 tag at N-terminal. Negative control samples in (**a**) consist of total cell protein samples from BL21 cells that were transformed with pET empty vector. The uncropped original gels are presented in Supplementary Fig. S1.

Different amounts of purified proteins varying between 2 and 3 µg/µL were obtained: 2.5 µg/µL for P537 and 3 µg/µL and 2 µg/µL for P153 and P264, respectively. Despite several attempts to optimize protein expression for P2091, P509, and P561, very low amounts of these expressed proteins were obtained and the presence of other contaminants also eluting with the 10 mM imidazole concentration used prevented us from measuring concentrations specific to these recombinant proteins. Transmembrane regions-encoding sequences were excluded from P561 gene sequence that led to improving the expression yield to 17 µg/µL. Even after the deletion of the transmembrane region in P561, efforts to purify this candidate under native conditions failed. Different strategies, including decreasing the concentration of IPTG and reducing culture temperature in the incubation phase, were tested to overcome this challenge, but since they were unsuccessful, the purification of this candidate was conducted under denaturing conditions. For financial and time reasons, the synthesis and cloning of the truncated form of P509 as well as the optimization of the expression of P2091 through the cloning of its truncated form were not considered in the current work. As the cloning of the truncated form of P509 repetitively failed, the full-length form of the protein was used to immunize birds, although the solution contained other contaminants (Fig. 1). The protein concentration of the whole-cell lysate obtained from *C. perfringens* MLG_7820 was 14.6 µg/µL. The name, size, location, and VaxiJen score of the proteins that were produced and used for the immunization trial are presented in Table 1.

### Western blot analysis of recombinant proteins

**Figure 2:**
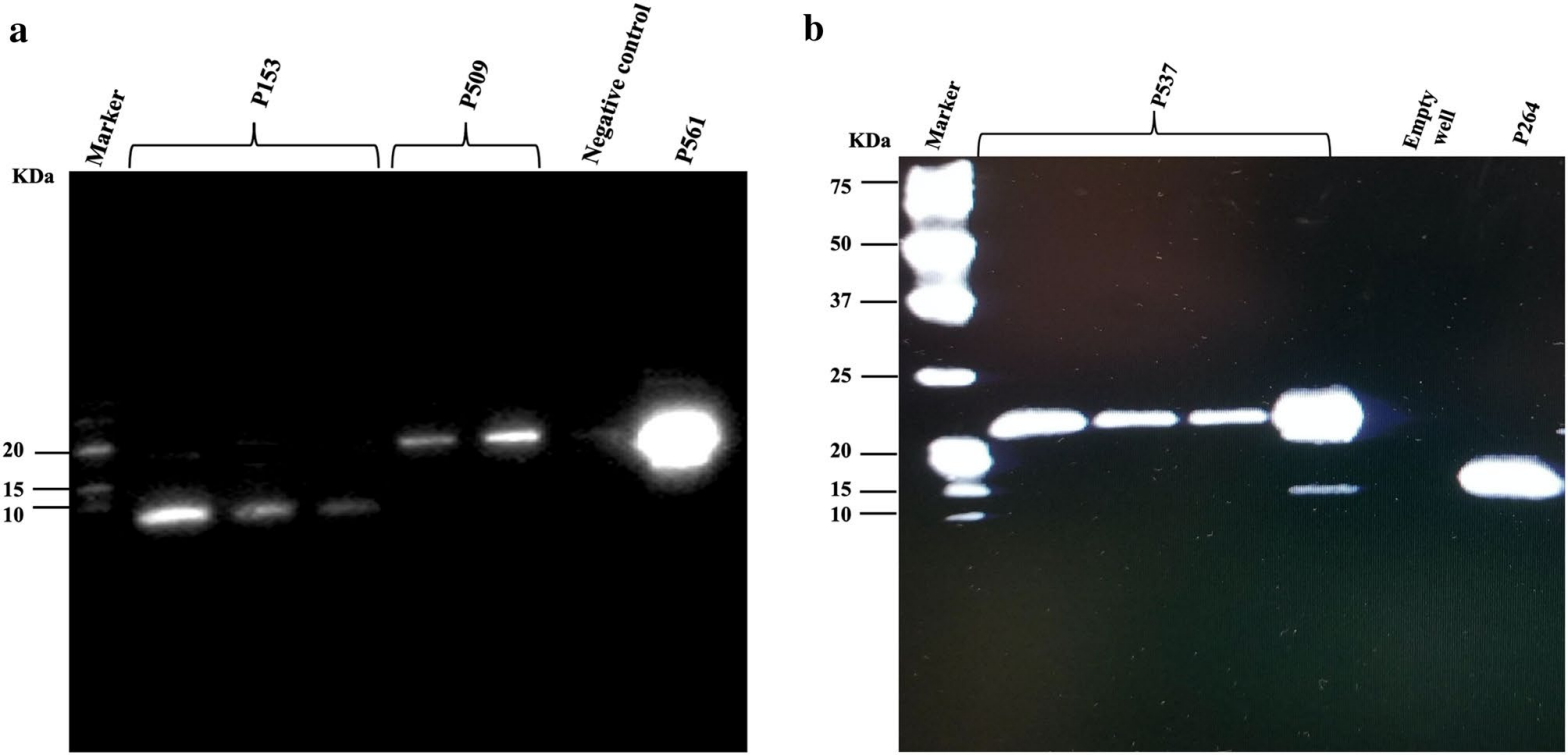
Western blot analysis of V5-tagged purified recombinant proteins: (**a**) P153, P509 (different fractions), Negative control, and P561 and (**b**) P537 (different fractions) and P264. Negative control sample in (**a**) consist of total cell protein samples from BL21 cells that were transformed with pET empty vector.

**Figure 3:**
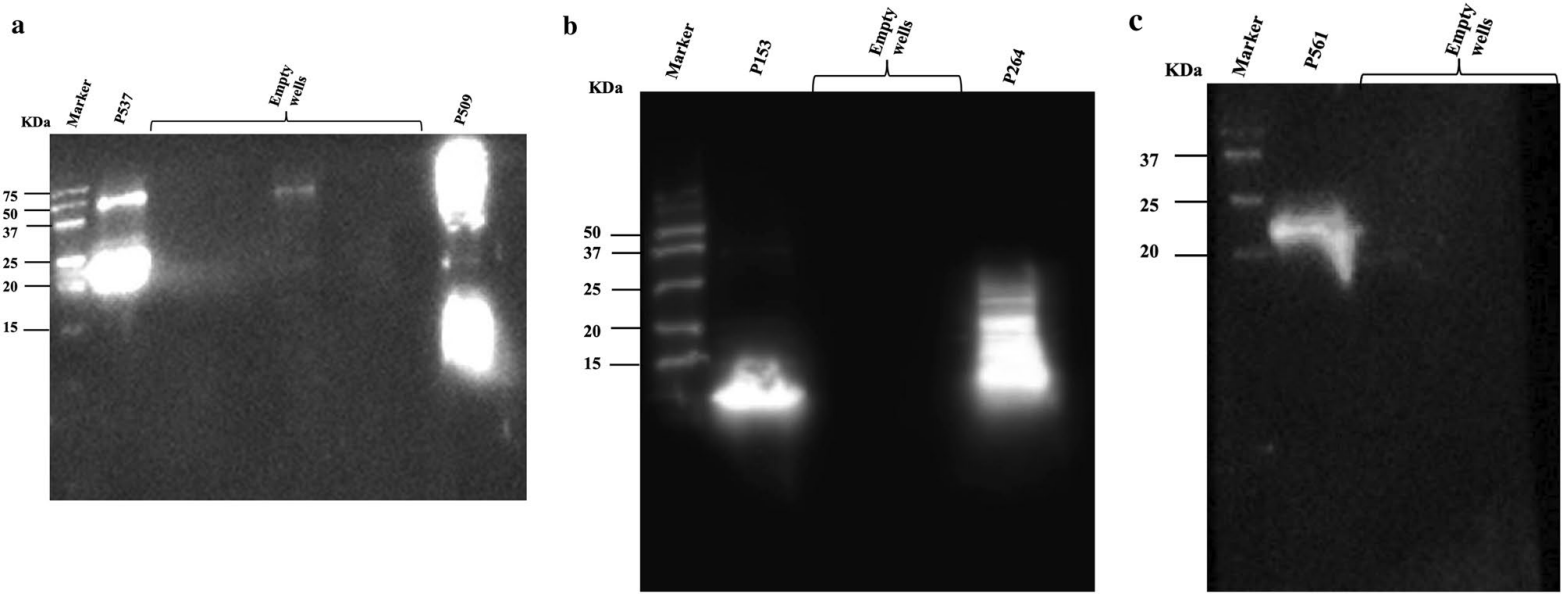
Western blot analysis of chicken antibodies raised against recombinant forms of (**a**) P537 and P509, (**b**) P153 and P264, and (**c**) P561.

Results of Western blotting indicated that the eluted purified proteins were detected by Anti-V5-HRP Antibody (Fig. 2). Serum samples collected from vaccinated birds at day 35 revealed the presence of antibodies able to recognize their respective recombinant protein (Fig. 3).

### Measurement of serum antibody levels by ELISA

Statistical analyzes showed a significant interaction between time and candidate proteins (GLM: P-value < 0.001), indicating different responses over time between different candidates. ELISA results evaluating antibody levels showed significant differences in the serum antibody response (Post-hoc tests: P-values < 0.001) on days 14, 21, and 35 in comparison to day 7 for the birds immunized with P537. A similar trend was also observed for birds from group P264 (Post-hoc tests: P-values < 0.01), whereas a third immunization was required to significantly increase antibody response in birds immunized with the other candidate proteins. Globally, the fold-change in IgY titers between day 7 and day 35 in immunized birds varied from 8.8, 16.8, 23.8, 53 and 9110 for the P153, P561, P264, P509 and P537 groups, respectively. The highest mean IgY titers were documented in birds from groups P509 (8.28E + 04 and 2.41E + 05) and P537 (5.08E + 05 and 9.11E + 05) on days 21 and 35, respectively. Birds from these two groups however presented with distinct fold changes in IgY titers between day 7 and day 35, this fold-change being more than 170 greater for the birds immunized with P537. The average IgY antibody titers on days 21 and 35 were the lowest in birds immunized with P153 and P561, although a significant difference was noted between these days and day 7 and 14 in birds from groups P153 and P561, respectively (Post-hoc tests: P-values < 0.01).

**Figure 4:**
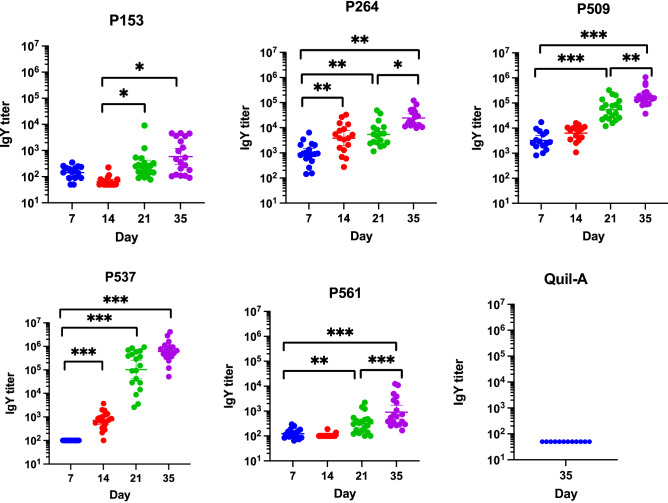
Evaluation of the serum IgY antibody titer on days 7, 14, 21, and 35 against the indicated recombinant protein by ELISA. Chickens were immunized intramuscularly with purified recombinant protein mixed with the adjuvant Quil-A. Birds from the negative control group were immunized with adjuvant alone. The error bars represent standard error on means of two repeats. *P ≤ 0.05; **P ≤ 0.01; ***P ≤ 0.001, determined by the likelihood ratio test (LRT), followed by a post-hoc Tukey test with a Benjamini–Hochberg correction.

The evaluation of the antibodies raised in immunized birds using ELISA confirmed their ability to recognize the recombinant form of their related candidate protein (Fig. 4). ELISA results revealed IgY antibody titers showing fold changes ranging from 8.8 to 9110 between the first immunization and the end of the trial at 35 days of age, with IgY antibody titers varying from 1.0E + 02 to 4.54E + 03 at 7 days of age and from 1.47E + 03 to 9.11E + 05 at time of slaughter, an increase that was shown to be statistically significant for all the candidate proteins evaluated (P153, P509, P264, and P561 (Post-hoc tests: P-values < 0.01), P537 (Post-hoc tests: P-values < 0.001)). The IgY titers documented in birds from the Quil-A control group at day 35 were inconsequential.

**Figure 5:**
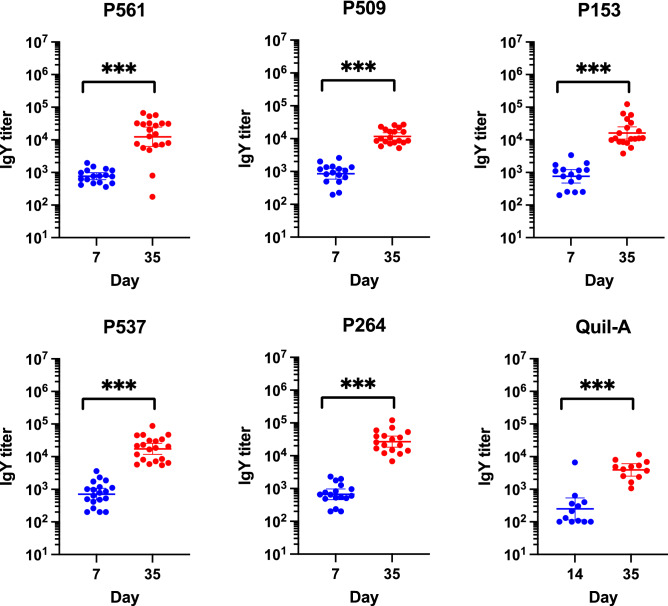
ELISA were performed with whole-cell lysate from *C. perfringens* MLG_7820 and P561, P509, P153, P264 and P537 antibodies raised in immunized birds. Quil-A response is also included. Immuno plate MaxiSorp wells were coated with 0.5 µg of whole-cell lysate from *C. perfringens* MLG_7820. The error bars representing standard error. The asterisks refer to the level of significance: *P ≤ 0.05; **P ≤ 0.01; ***P ≤ 0.001, determined by the Mann–Whitney test.

To document the capacity of the IgY antibodies raised in birds immunized with the recombinant form of the different candidate proteins to recognize the native forms of these proteins, the same ELISA approach was used, except that wells of the ELISA plate were coated with the whole cell lysate prepared from *C. perfringens* MLG_7820. IgY antibody titers varied from 8.5E + 02 to 1.05E + 03 for the different groups at day 7 (which was considered as non-specific background), whereas these same titers showed minimum and maximum values of 1.34E + 04 and 3.5E + 04 at day 35 (Fig. 5). According to the group, titers showed fold changes ranging from 12.8 to 41.2 between days 7 and 35. Regarding the Quil-A group, the average IgY antibody titers on days 14 and 35 were 7.64E + 02 and 4.75E + 03, and even if a slight increase was shown, it revealed not to be significant (Wilcoxon: P-value < 0.001). Average IgY antibody titers at day 35 measured from the birds immunized with the different candidate proteins were all found to be significantly higher when compared to the titers generated in birds receiving Quil-A (Wilcoxon: P-values varying from < 0.001 to 0.007 (Table 2).

**Table 2:** Comparison between the antibody titers of Quil-A control group and groups immunized with the candidate proteins, on day 35, against the whole cell lysate of *C. perfringens* MLG_7820, and their respective p-values.

Compared groups	Adjusted P-value (Bonferroni method)
Quil-A Vs. P153	0.007
Quil-A Vs. P264	0.001
Quil-A Vs. P537	< 0.001
Quil-A Vs. P509	0.001
Quil-A Vs. P561	0.007

## Discussion

NE remains a major constraint on the transition-away from routine use of AGPs for the poultry industry worldwide and an effective vaccine providing full protection against the disease is yet to be commercialized^33,34^. The main objective of the current work was to assess the immunogenicity of five candidate proteins identified from six distinct NE-causing strains of *C. perfringens* using a CSRV approach. Among the five tested antigens in this study, P509 demonstrated the highest average antibody titer (2.41E + 05). This protein is predicted as a prepilin N-terminal cleavage methylation domain protein which is suspected to form the subunits of a pilus structure, an external hair-like appendage in bacteria such as *C. perfringens*^35^. The role of pili in the pathogenesis of NE has been more recently brought to light with a genetic signature being recognized in NE-causing *C. perfringens* strains, this signature comprising a chromosomal locus called VR-10B and encoding a sortase-dependent pilus shown to contribute to the adhesion and disease-causing ability of virulent *C. perfringens*^13,23,36,37^. Pilin-encoding sequences usually include a signal peptide containing a typical prepilin peptidase cleavage site, harbor an N-terminal transmembrane domain and are relatively small^35,38^. When working with pilin-encoding sequences, other authors reported issues with cloning as well as poor levels of expression^23,39^. The presence of a hydrophobic transmembrane signal peptide at the N-terminal has been pointed out as the major interfering element and might have contributed to the difficulty encountered in the cloning and expression of P509 and P561, which are both prepilin N-terminal cleavage methylation domain proteins. It should be pointed out that P561 was the only protein that was purified under denaturing conditions in order to improve its solubility, and this denaturation might have acted as a contributing factor to the low levels of specific IgY antibodies generated in immunized birds^40,41^. However, as P561 was identified as a prepilin N-terminal cleavage methylation domain protein that could both be part of a functional pilus found on the surface of the pathogenic *C. perfringens* strains analyzed and contribute to the development of a protective immunity in the avian host owed to its localization, the magnitude of the IgY antibody titers raised should be correlated with the protection capacity of these antibodies^23,42^. When presenting the gene organization and structural modelling of type IV pili (T4P) in the reference strain *C. perfringens* 13, Melville and Craig^43^ not only suggested the presence of T4P in most *Clostridia* species, but also highlighted the differential contribution of the different components to the framework of this adhesion structure, with pilin subunits acting as either minor or major pilins^43^. Thus, whether acting as a major pilin forming the structure of the pilus itself, or as a minor pilin contributing in most part to the function of the structure, we can hypothesize that P509 and P561 proteins, according to their role and localization, might not be equally exposed to the immune system of the  carrying avian host harboring *C. perfringens* and could then be at the root of the significant difference in the basal levels of immunity observed in birds from groups P509 and P561 at both day 7 and at day 35. Even though different characteristics can influence the immunogenicity of a specific antigen, results from the current study revealed that the immunogenic score predicted in silico might not be the most reliable criterion when it comes to selecting suitable immunogenic targets. This affirmation is especially true regarding the IgY antibody titers raised in birds immunized with P537, a candidate protein that was included from the first steps of the CSRV pipeline and used during the in vivo assay as a weak antigen to which the other candidate proteins would be compared for the immunogenic potential. Indeed, P537 was predicted as a weakly antigenic protein, with a VaxiJen score of 0.26. Surprisingly, this candidate was associated with the highest IgY titers observed in immunized birds (mean IgY titer of 9.11E + 05 at day 35), in addition to inducing the greatest fold change in these titers between the first and last blood collection. Apart from being the candidate protein with the lowest predicted immunogenic score, P537 was also selected on the basis of its localization that was predicted to be cytoplasmic^32^. This localization inside the *C. perfringens* bacterial cell might explain why the birds vaccinated with this candidate showed the lowest basal level reaction against this protein at day 7 (mean IgY titer of 1.0E + 02). Indeed, prior to the first immunization, basal IgY response against P537 were significantly lower when compared to the basal titers against P264 and P509 proteins, candidate proteins for which an extracellular localization was predicted (see Table 1). This observation again emphasizes the point that P561 protein might play a functional rather than a structural role in the pilus structure to which it was predicted to contribute, as basal IgY levels at day 7 against the recombinant form of this protein were similar to those against the cytoplasmic protein P537 identified as a rubrerythrin, a non-heme iron protein that is involved in the bacterial response to oxidative stress conditions^44,45^. The same observation holds true for the results observed at day 7 for the basal levels of IgY against P153, a candidate protein for which consensus could not be reached by the prediction tools used regarding its cellular localization that was then predicted as either cytoplasmic or extracellular^32^. The intracellular localization of P153 might have played a role in the significant differences observed in the basal levels of immunity at 7 days of age when compared to those observed for P264 and P509, for which the localization of the candidate protein was predicted to be extracellular. However, the particularly low molecular weight (5 kDa) of P153 might have more importantly contributed to the significantly weaker active immune response induced after the injection of 3 doses in birds immunized with this candidate when compared to the response generated in birds injected with P264. It is well-known that antigens for which the molecular weight falls below 10 kDa are less competent at stimulating the immune system^46–50^. However, despite a low molecular weight or a localization that was not predicted to be bacterial cell surface for some of the candidate proteins, as well as the inconsistency in antigen delivery based on the distribution of IgY titers in some groups, the current study showed that all the proteins tested were able to elicit a significant immune response after only two doses (Fig. 4), an immunization scheme that was reported by Lepp et al. as suboptimal when evaluating the immunostimulatory capacity of other adhensins of *C. perfringens*^23^. For the P264, P509 and P561 candidate proteins, a third dose was however associated with an improved immune response, whereas a single dose already elicited a significant increase in IgY titers for birds immunized with P264 and P537 recombinant proteins (see Fig. 4). Even though these observations should be correlated with the protection capacity of the raised antibodies in NE-challenged birds, the ability of the selected candidate proteins to elicit an early immune response make them attractive as vaccine targets that could confer immunity to broiler chickens before they become susceptible to NE in commercial conditions^51^. Protection assays should then be conducted to validate this potential before alternative delivery routes compatible with an application in the field can be considered. In the current study, the specificity of the antibodies raised in birds immunized with the different candidate proteins was evaluated^52^. Both ELISA and Western blotting results support the evidence for an immune response in immunized birds that is specifically raised in response to the recognition of a foreign stimulus, this recognition being translated into a significant increase in IgY titers between day 7 and day 35 in serum samples analyzed against the recombinant form of all the candidate proteins evaluated (Fig. 4). The capacity of these raised antibodies to recognize the native proteins in the whole bacteria was also documented using a whole cell lysate from *C. perfringens* MLG_7820 and the results obtained again point out to a similar conclusion (Fig. 5). It is however remarkably important to highlight the fact that even though various studies reporting the immunogenic potential of some *C. perfringens*-derived antigens used Quil-A as an adjuvant to induce both cell and humoral-mediated immune responses in avian birds, a very limited number of these studies documented the magnitude of this Quil-A-induced immune response in this host^21–23,53–55^. When evaluating the protective capacity of a recombinant form of the *C. perfringens* alpha toxin against a NE challenge in broiler chickens, Cooper and Songer reported titers twofold higher for birds injected with Quil-A at 5 and 15 days of age, when compared to the negative control group^55^. Similarly, Jiang et al. conducted various trials comparing the immunogenic properties of two recombinant proteins, an endo-beta-N-acetylglucosaminidase and a phosphoglyceromutase from NE-causing *C. perfringens* and reported fold changes ranging from 1.7 and 3 in IgY titers between broiler chickens used as a negative control and birds injected with the Quil-A adjuvant. Additionally, the magnitude of this increase in IgY titers at day 24 between groups receiving Quil-A and groups immunized with the recombinant form of the proteins tested was 3.0 and 2.8 for trials 1 and 2, respectively, after immunizations performed at 7, 14 and 21 days of age^21^. In the current study, even though the capacity of the raised antibodies to recognize the native protein fare less well than the abilty of these same antibodies to specifically bind to the recombinent form of the candidate proteins, the IgY titers obtained through the ELISA approach using the *C. perfringens* MLG_7820 whole cell lysate showed fold changes varying between 2.8 and 7.4 when comparing the mean IgY titers from the Quil-A-injected birds and the groups immunized with the different candidate proteins.

Taken together, findings of the current study emphasize the usefulness of a CSRV approach for the rapid identification of putative vaccine candidates, and to our knowledge, this is the first study in searching for surface-exposed vaccine candidate proteins among NE-causing *C. perfringens* using CSRV. The immunogenic properties of these vaccine candidates are promising and assessment of their protective ability using a NE experimental challenge model should be done.

## Materials and methods

### Bioinformatics analyses

The immunogenicity of the vaccine candidate proteins was predicted earlier^32^ using the Vaxijen v2.0 tool that predicts protective antigens and subunit vaccine candidates based on their protein sequence (http://www.ddg-pharmfac.net/vaxijen/VaxiJen/VaxiJen.html) with a default threshold of 0.5. In other words, candidates with a VaxiJen score below 0.5 were considered non-antigenic and therefore excluded from further analysis. For each candidate protein, the GRAVY score which represents the hydrophobic nature of the protein was calculated (https://www.bioinformatics.org/sms2/protein_gravy.html). Proteins with a GRAVY score above 0 are considered hydrophobic in nature, while a GRAVY score below 0 indicates a hydrophilic protein. The prediction of both signal peptides (https://services.healthtech.dtu.dk/service.php?SignalP-5.0) and transmembrane regions (https://services.healthtech.dtu.dk/service.php?TMHMM-2.0) was also made. A BLAST search was done to compare the resultant sequences with in silico identified protein-encoding genes. One candidate with a low predicted antigenic score was included as a negative control to validate in vivo the reverse vaccinology pipeline used in this paper.

### Validation of in silico studies, amplification, and cloning of candidate protein-encoding genes

To validate in silico studies, forward and reverse primers were designed for each candidate protein-encoding gene. The list and sequence of primers used are presented in Table 3. Genomic DNA was extracted from a virulent *C*. *perfringens* strain from our collection, MLG_7820, by the Chelex DNA extraction method. Briefly, *C*. *perfringens* MLG_7820 was grown overnight on 5% sheep blood agar plates (Fisher, Ottawa, ON) at 37 °C under anaerobic condition (Oxoid AnaeroGen gas packs (Thermo Fisher Scientific, MA, USA)). Seven to ten colonies were suspended in 1 mL of ddH_2_O, followed by centrifugation at 10,000×*g* for 2 min (Eppendorf 5415C centrifuge). A 200 μL volume of a 10% Chelex® 100 solution (Bio-Rad, CA, catalog no. 143- 2832) was added to the pellet and samples were vortexed before being heated at 56 °C for 30 min (Dry Bath Incubator, Fisher Scientific) with a subsequent boiling step in water for 10 min. The DNA was collected after centrifugation at 13,000×*g* for 2 min and used for the amplification of the candidate protein-encoding genes by PCR. A unique PCR program was used for all genes and cycling conditions were as follows: a hot start step of 2 min at 95 °C, followed by 30 cycles of 20 s at 95 °C, 20 s at 41.3 °C, and 2 min at 72 °C, with a final elongation step of 10 min at 72 °C. A volume of 5 μL of each PCR product was analyzed by migration on a 1% (w/v) agarose gel containing 0.01% SYBR Safe DNA gel stain (Fisher, Ottawa, ON), followed by visualization under UV light and using a TrackIt 1 Kb Plus DNA ladder (Invitrogen, Carlsbad, CA). Sequencing of PCR products was conducted at McGill University (Québec, Canada) and Génome Québec Innovation Centre (Montréal, QC, Canada), before cloning into pET151/D-TOPO vector using *E. coli* TOP10 cells (Champion pET151 Directional TOPO Expression Kit, Invitrogen, CA, USA) according to the manufacturer’s instructions. Ten colonies were picked up randomly and analyzed by colony PCR to screen for transformants that contained the recombinant plasmid. Briefly, single colonies were transferred to new LB agar plates supplemented with 100 μg/mL ampicillin (Fisher Scientific New Jersey, USA), followed by overnight incubation at 37 °C. One loop of bacterial cells was collected and suspended in 1 mL of ddH_2_O, followed by vortex and centrifugation at 10,000×*g* for 3 min. A 200 μL volume of 10% Chelex® 100 was added to the pellet, with a subsequent vortex and boiling in water for 10 min. Samples were then centrifuged at 10,000×*g* for 2 min and 3 μL of supernatant was used as a template for PCR amplification using the same primers and PCR program described above. Positive transformants were sent for direct colony sequencing to the Centre Hospitalier Universitaire de Québec-Université Laval Research Center (Québec, Canada). Recombinant plasmids were extracted using the QIAprep Spin Miniprep Kit (Qiagen, Hilden, Germany, catalog no. 27106) according to the manufacturer’s instructions. For the candidate protein P509, synthesis and cloning into pET-24a(+) vector was done by GenScript (Piscataway, NJ, USA). To do that, the protein-encoding gene was first synthesized by Oligo Synthesizer (Biolytic Lab Performance, Inc. Fremont, USA) and then cloned into the vector using *Bam*HI and *Nde*I enzymes.

**Table 3:** Selected candidate proteins with their respective primer sequences and amplicon size after PCR amplification.

Name of candidate	Primer sequence (5’–3’)	Amplicon size (bp)
Hypothetical protein	Forward CACCTTGTTAAATATAGATAGA Reverse TTATTTATTCTTACAACAACCA	264–2
Hypothetical protein	Forward CACCATGAACAAGAACATGGAA Reverse TTATTTATCAAATAATCCTCCA	153
Prepilin N-terminal cleavage methylation domain	Forward CACCGATGTAGCAAGTGCTAAA Reverse TATTAATGACTTGCTGGTGTTG	509
Prepilin N-terminal cleavage methylation domain	Forward CACCAATTATGTAACTTTAGAT Reverse TTATTTTTTATTTCTAAAAGTT	561
Rubrerythrin (low antigenic negative control)	Forward CACCATGAAAAGTTTAAAAGGA Reverse CTAACATTCATTTAAAAGTCTA	537
Sensor Histidine Kinase	Forward CACCTTGGCTATAAAATCGAAA Reverse TTACTTTTTCTTAAAGCTTATT	2232
Formate/nitrite transporter family protein	Forward CACCATGCAAAAAAAAGATATA Reverse TTATTCCATGCTCATAAATTTA	759
FAD-dependent oxidoreductase	Forward CACCATGAGAGAATATGATTTA Reverse TTAACTTATAGTTTTAATAAGC	891
Preprotein translocase YajC	Forward CACCATGAAAACTTTTATGATT Reverse TTATAAAACCTTAGAGACACAA	264–1
SPOR domain-containing protein	Forward CACCGTGAAGTACACTAGAATT Reverse TTATTTGTTTACTTTAAAATTA	804
Isopeptide-forming domain-containing fimbrial protein	Forward CACCATGAAAAATAATATATTA Reverse TTAGTTCTTAGTTTTTCTGCTA	1569
SdpI family protein	Forward CACCATGGGATTTTGGATTTTT Reverse CTACACATTTTTTCTATTGCCA	384
Cna B-type domain-containing protein	Forward CACCATGAAAATAAATAAAAAA Reverse TCATCTTTTCAGTCCTTTATTC	2091
Pilus backbone structural protein	Forward CACCATGATAAACAAGAAAAAA Reverse TTATCTTATAGTTTTACGTCTT	1074

### Expression and purification of candidate proteins

Ten ng of plasmid DNA was transformed into BL21 Star™(DE3) One Shot® cells via a heat-shock method according to the manufacturer’s instructions (Invitrogen, CA, USA, catalog no. K151-01). One single transformed colony was grown in 30 mL of Luria Bertani broth (BD Difco, MD, USA, catalog no. DF0446-07-5) supplemented with 100 μg/mL ampicillin (Fisher Scientific New Jersey, USA) for 17 h, at 37 °C with shaking (180 rpm). The overnight culture was then added to 600 mL Luria Bertani broth (supplemented with 100 μg/mL ampicillin) and incubated for 2 h at 37 °C with shaking (180 rpm) until an OD_600_ of 0.6 was reached. The expression of recombinant proteins was induced by the addition of isopropyl β-d-1-thiogalactopyranoside (IPTG) (Invitrogen, CA, USA, catalog no.15529019) to a final concentration of 1 mM. After five hours of incubation, all cultures were harvested by centrifugation at 3000×*g*, 4 °C for 20 min (Thermo Scientific Sorvall Legend XTR with F14-6 × 250y Fixed-Angle Rotor). The pellet was preserved at − 20 °C until purification. Purification of all proteins, except for P561, was conducted under native conditions since they were all soluble proteins. To do so, the stored pellet was re-suspended in 20 mL of lysis buffer (50 mM NaH_2_PO_4_, 300 mM NaCl, 10 mM imidazole, pH 8) supplemented with 1X protease inhibitor cocktail (Roche, Mannheim, Germany), and lysozyme ((1 mg/mL) Thermo Scientific, MA, USA, catalog no. J60701), followed by sonication on ice using a sonicator with a microtip probe (Ultrasonic processor, model: GE130). A protocol involving six 10 s bursts at 250 W with 10 s cooling intervals between each burst was used. The lysate was then centrifuged (15,000×*g*, 4 °C, 20 min (Thermo Scientific Sorvall Legend XTR with Fiberlite™ F15-8 × 50cy Fixed Angle Rotor)) and the supernatant was loaded onto a 5 mL HisTrap HP column (GE Healthcare, Montreal, CA, catalog no. 17-5248-02) pre-equilibrated with the same lysis buffer mentioned above. His-tagged proteins were purified using a FPLC ÄKTA-purifier system (GE-Healthcare, Uppsala, Sweden) and a 50–500 mM imidazole gradient (Sigma-Aldrich, USA, catalog no. 792527). One mL fractions were collected, and since proteins showed strong absorption in the ultraviolet (UV) region at UV 280 nm, fractions showing a 280 nm peak were subjected to SDS-PAGE. Samples containing the protein of interest were desalted using PD-10 desalting columns packed with Sephadex G-25 resin (GE Healthcare, USA, catalog no. 17-0851-01). For the purification of P561 under denaturing conditions, the pellet was resuspended in 20 mL of lysis buffer (100 mM NaH_2_PO_4_, 10 mM Tris·Cl, 8 M urea, pH 8) and stirred (Barnstead Thermolyne, Roto Mix-Type 50800) for 60 min at room temperature before being sonicated as described above. The lysate was centrifuged at 10,000×*g* for 30 min at room temperature (Thermo Scientific Sorvall Legend XTR with Fiberlite™ F15-8 × 50cy Fixed Angle Rotor) and the supernatant was loaded onto a 5 mL HisTrap HP column in the ÄKTA-purifier system. The protein of interest was eluted with an elution buffer (100 mM NaH_2_PO_4_, 10 mM Tris·Cl, 8 M urea, pH 4.5), after washing the column with wash buffer (100 mM NaH_2_PO_4_, 10 mM Tris·Cl, 8 M urea, pH 6.3). Individual fractions were collected, analyzed by SDS-PAGE, and desalted using PD-10 desalting columns. Quantitation of the purified candidate proteins was performed using the Qubit Protein Assay kit (Invitrogen, CA, USA, catalog no. Q33211) and Denovix QFX Fluorometer (Froggabio, Toronto, ON), according to the manufacturer’s instructions. Aliquots of proteins were stored at -80 °C until used in the in vivo assays.

### Preparation of whole-cell lysate from *C. perfringens* MLG_7820

*C. perfringens* cells were grown at 37 °C in 30 mL of a medium made of 25% nutrient broth (EMD Chemicals Inc., Gibbstown, NJ), 50% tryptic soy broth (BD Biosciences, Franklin, NJ), and 25% peptone water (Biokar Diagnostics, France) under anaerobic conditions. The resulting culture was harvested after 24 h and cells were pelleted by centrifugation (5000×*g*, 4 °C, 20 min (Thermo Scientific Sorvall Legend XTR with Fiberlite™ F15-8 × 50cy Fixed Angle Rotor)). The recovered pellet was then resuspended in 5 mL of phosphate-buffered saline (PBS), followed by eight repeated freeze–thaw cycles in liquid nitrogen^56^. The protein concentration was measured (Qubit Protein Assay kit and Denovix QFX Fluorometer), and aliquots of proteins were stored at − 80 °C until the vaccination trial.

### Birds housing and feeding

This project was accepted and carried out under the guidelines of the Animal Ethics Committee of the Faculté de Médecine Vétérinaire of the Université de Montréal (certificate #21-Rech-2070) and under the ARRIVE guidelines^57^. The in vivo study was approved by the Comité d'Éthique sur l'Utilisation des Animaux (CÉUA) of the Faculté de Médecine Vétérinaire of the Université de Montréal (certificate number: 20-Rech-2070). In this study, 140 commercial day-old male Ross broiler chickens vaccinated against Marek’s disease and avian bronchitis were bought directly from a commercial hatchery and were randomly divided into seven experimental groups (n = 20): (1) Adjuvant-only (Quil-A), (2) P561, (3) P509, (4) P537, (5) P153, (6) P264 and (7) Hyperimmunized group (whole-cell lysate from *C. perfringens* MLG_7820). Over a period of 35 days, birds were fed a commercial diet void of any antibiotics and anticoccidials (Meunerie Benjamin, St-Césaire, Québec, Canada) and corresponding to the starter, grower, and finisher stages of a regular commercial broiler diet. Birds were euthanized at 35 days of age using cervical dislocation with prior sedation with 0.8 mL/kg of a xylazine (100 mg/mL)/ketamine (100 mg/mL) blend.

### Preparation of recombinant proteins and immunization of broiler chickens

All recombinant proteins were prepared early in the morning. Briefly, proteins were thawed on ice and the concentration of each candidate was measured using the Qubit Protein Assay kit (Invitrogen, CA, USA, catalog no. Q33211) and Denovix QFX Fluorometer (Froggabio, Toronto, ON), according to the manufacturer’s instructions. Each bird was immunized intramuscularly in the pectoral muscle with 200 μL of PBS containing each recombinant protein (50 μg) and Quil-A adjuvant ((50 μg) InvivoGen, CA, USA) at days 7, 14, and 21 days of age. Birds from the hyperimmunized group were vaccinated with 200 μL of PBS containing 50 μg of proteins from the whole cell lysate preparation and Quil-A adjuvant (50 μg). Birds from the adjuvant control group received 50 μg of Quil-A in a 200 μL volume of PBS. Blood samples were collected from all birds prior to each immunization and at day 35 (end of the trial) in blood collection tubes (Covidien, Monoject blood collection tube, MA, USA, catalog no. 8881301215). Blood tubes were maintained at room temperature for 3 to 4 h and then centrifuged (3000 rpm, 19 °C, 10 min, Beckman Coulter with SX4750A Rotor) before the serum was collected. Serum samples were stored at − 20 °C until analysis by ELISA.

### Western blot analysis of recombinant proteins

Recombinant proteins were separated on a 15% gel using an SDS-PAGE approach under reducing conditions, followed by a transfer onto a PVDF membrane (BIO-RAD, CA, USA, catalog no.1620177) at 100 V for 1 h in 1X transfer buffer (48 mM Tris, 39 mM glycine, 20% methanol, 0.1% SDS). The membrane was blocked with blocking buffer (5% skim milk, 1% TBS, 0.1% Tween-20) with gentle agitation for 45 min at room temperature. The membrane was then incubated with StrepTactin-Horseradish Peroxidase (HRP) conjugate (Bio-Rad, USA; (previously diluted 1/5 in ddH_2_O and added at a ratio of 2:15,000 in blocking buffer)) to detect the protein ladder (Precision Plus Protein WesternC standards; Bio-Rad, USA), and Anti-V5-HRP Antibody (Invitrogen, USA; (at the same proportion described above)) to identify the protein of interest, with shaking for 1 h at room temperature.

To investigate whether the raised antibodies in broiler chickens can recognize the recombinant form of the candidate proteins, the generated antibodies in birds (diluted 1:200 in blocking buffer) and goat anti-chicken IgY horseradish peroxidase (HRP)-conjugated polyclonal antibody (Bethyl Laboratories, Montgomery, TX, USA; (diluted 1:5000 in blocking buffer)) were submitted to the same procedure explained above as the primary and secondary antibodies, respectively, with one hour of incubation at room temperature for each step. A chemiluminescent detection was performed with Clarity Western ECL Substrate (BIO-RAD, CA, USA, catalog no. 1705060) according to the manufacturer’s instructions (Fusion FX imaging system (Vilber Lourmat; SU, Germany)).

### Measurement of serum antibody levels by ELISA

Antibody titers against the recombinant proteins as well as the whole-cell lysate proteins of *C. perfringens* MLG_7820 were analyzed by ELISA. To do that, the recombinant purified proteins or whole-cell lysate proteins of *C. perfringens* MLG_7820 were diluted to 5 μg/mL in 50 mM carbonate/bicarbonate coating buffer (pH 9.6), and 100 μL of this dilution was added to each well of a 96 well flat-bottom immuno plate MaxiSorp (Thermo Scientific, NY, USA, catalog no. 475094). Plates were then incubated for 1 h at 37 °C, followed by an overnight incubation at 4 °C. After being washed three times with washing buffer (phosphate-buffered saline [PBS] containing 0.05% Tween 20), plates were blocked (blocking buffer made of PBS containing 0.05% Tween 20 and 0.3% casein (Sigma-Aldrich, USA, catalog no.C7078)) and incubated for 2 h at 37 °C. The plates were washed again three times with the same washing buffer before all the wells were coated with 100 μL of serum sample, using a two-fold serial dilution series (ranging from 1:50 to 1: 3,276,800) in blocking buffer. After incubation for 2 h at 37 °C and three washes with washing buffer, 100 μL of goat anti-chicken IgY horseradish peroxidase (HRP)-conjugated polyclonal antibody ((Bethyl Laboratories, Montgomery, TX, USA, cat. no A30-104P); diluted 1:8000 in wash buffer) was added to each well, followed by an incubation step at 37 °C for 1 h and three washes (wash buffer). Subsequently, 100 μL of 3,3′,5,5′-tetramethylbenzidine (TMB; Life Technologies, Inc., CA, USA) substrate was applied to each well and plates were incubated in the dark at room temperature. The absorbance was measured periodically at 650 nm until the OD of the positive control (serum collected from birds immunized with the whole cell lysate from *C. perfringens* MLG_7820) reached 0.45. At this point, 100 μL of 0.18 M H_2_SO_4_ was added to each well and the final OD reading was taken at 450 nm by a spectrophotometer (EZ Read 400, Biochrom, UK), according to the manufacturer’s instructions.

### Statistical analysis

In this study, data distributions were not normal (Shapiro's tests < 0.05) but were rather of the Gamma type. Consequently, we used generalized linear models (GLMs) with Gamma family for statistical analysis of ELISA results generated from serum samples collected at days 7, 14, 21, 35 days of age. Results were then extracted via likelihood ratio tests (LRTs). Tukey’s post-hoc tests with Benjamini–Hochberg correction on the P-values was carried out due to multiple comparisons. A Mann–Whitney test was used for ELISA results obtained from the analysis of serum samples on days 7 and 35 against the whole cell lysate of *C. perfringens* MLG_7820. The non-parametric Wilcoxon test was used to compare the antibody titers of Quil-A control group on days 14 and 35 against the whole cell lysate of *C. perfringens* MLG_7820 as well as the comparison between the antibody titers of Quil-A and vaccinated groups on day 35 against the whole cell lysate of *C. perfringens* MLG_7820, followed by the Bonferroni correction due to multiple comparisons. All the statistical analyzes were carried out with the software R Version 4.0.3 (R Core Team, 2020)^58^ and the significance α threshold was set at 0.05.

## Supplementary Information

Supplementary Information.

## Data Availability

Data are available in the SRA database as project ID: PRJNA734442.

## References

[1] M'Sadeq SA, Wu S, Swick RA, Choct M (2015). Towards the control of necrotic enteritis in broiler chickens with in-feed antibiotics phasing-out worldwide. Anim. Nutr..

[2] Hardy SP (2020). Developing an experimental necrotic enteritis model in turkeys-the impact of *Clostridium perfringens*, *Eimeria meleagrimitis* and host age on frequency of severe intestinal lesions. BMC Vet. Res..

[3] Rood JI (2018). Expansion of the *Clostridium perfringens* toxin-based typing scheme. Anaerobe.

[4] Kiu R, Hall LJ (2018). An update on the human and animal enteric pathogen *Clostridium perfringens*. Emerg Microbes Infect..

[5] Prescott JF, Smyth JA, Shojadoost B, Vince A (2016). Experimental reproduction of necrotic enteritis in chickens: A review. Avian Pathol..

[6] Abd El-Hack ME (2021). Necrotic enteritis in broiler chickens: Disease characteristics and prevention using organic antibiotic alternatives: A comprehensive review. Poult. Sci. J..

[7] Alizadeh M (2021). Necrotic enteritis in chickens: A review of pathogenesis, immune responses and prevention, focusing on probiotics and vaccination. Anim. Health Res. Rev..

[8] Keyburn AL (2013). Vaccination with recombinant NetB toxin partially protects broiler chickens from necrotic enteritis. Vet. Res..

[9] Fernandes da Costa SP (2016). Variable protection against experimental broiler necrotic enteritis after immunization with the C-terminal fragment of *Clostridium perfringens* alpha-toxin and a non-toxic NetB variant. Avian Pathol..

[10] Prescott JF, Parreira VR, MehdizadehGohari I, Lepp D, Gong J (2016). The pathogenesis of necrotic enteritis in chickens: What we know and what we need to know: A review. Avian Pathol..

[11] Lacey JA, Johanesen PA, Lyras D, Moore RJ (2016). Genomic diversity of necrotic enteritis-associated strains of *Clostridium perfringens*: a review. Avian Pathol..

[12] Emami NK, Dalloul RA (2021). Centennial review: Recent developments in host-pathogen interactions during necrotic enteritis in poultry. Poult. Sci. J..

[13] Lepp D (2021). *Clostridium perfringens* produces an adhesive pilus required for the pathogenesis of necrotic enteritis in poultry. J. Bacteriol..

[14] Shojadoost B, Vince AR, Prescott JF (2012). The successful experimental induction of necrotic enteritis in chickens by *Clostridium perfringens*: A critical review. Vet. Res..

[15] Wei B (2020). Antimicrobial susceptibility and association with toxin determinants in *Clostridium perfringens* isolates from chickens. Microorganisms.

[16] Mahmood K (2014). Non-antibiotic strategies for the control of necrotic enteritis in poultry. Poult. Sci. J..

[17] Mot D, Timbermont L, Haesebrouck F, Ducatelle R, Van Immerseel F (2014). Progress and problems in vaccination against necrotic enteritis in broiler chickens. Avian Pathol..

[18] Agency, E. M. *Netvax*. https://www.ema.europa.eu/en/medicines/veterinary/EPAR/netvax (2014).

[19] Wilde S (2019). *Salmonella*-vectored vaccine delivering three *Clostridium perfringens* antigens protects poultry against necrotic enteritis. PLoS ONE.

[20] Kulkarni R, Parreira V, Sharif S, Prescott J (2008). Oral immunization of broiler chickens against necrotic enteritis with an attenuated *Salmonella* vaccine vector expressing *Clostridium perfringens* antigens. Vaccine.

[21] Jiang Y, Kulkarni RR, Parreira VR, Prescott JF (2009). Immunization of broiler chickens against *clostridium perfringens*–Induced necrotic enteritis using purified recombinant immunogenic proteins. Avian Dis..

[22] Kulkarni R, Parreira V, Sharif S, Prescott J (2007). Immunization of broiler chickens against *Clostridium perfringens*-induced necrotic enteritis. CVI.

[23] Lepp D (2019). Immunization with subunits of a novel pilus produced by virulent *Clostridium perfringens* strains confers partial protection against necrotic enteritis in chickens. Vet. Microbiol..

[24] Kanampalliwar AM (2020). Reverse Vaccinology and Its Applications.

[25] Aldakheel FM (2021). Proteome-wide mapping and reverse vaccinology approaches to design a multi-epitope vaccine against *Clostridium perfringens*. Vaccines.

[26] Vivona S (2008). Computer-aided biotechnology: From immuno-informatics to reverse vaccinology. Trends Biotechnol..

[27] Masignani V, Pizza M, Moxon ER (2019). The development of a vaccine against *Meningococcus* B using reverse vaccinology. Front. Immunol..

[28] Viviani V, Biolchi A, Pizza M (2022). Synergistic activity of antibodies in the multicomponent 4CMenB vaccine. Expert Rev. Vaccines.

[29] Dalsass M, Brozzi A, Medini D, Rappuoli R (2019). Comparison of open-source reverse vaccinology programs for bacterial vaccine antigen discovery. Front. Immunol..

[30] Péchiné S, Bruxelle JF, Janoir C, Collignon A (2018). Targeting *Clostridium difficile* surface components to develop immunotherapeutic strategies against *Clostridium difficile* infection. Front. Microbiol..

[31] Wade B, Keyburn AL, Seemann T, Rood JI, Moore RJ (2015). Binding of *Clostridium perfringens* to collagen correlates with the ability to cause necrotic enteritis in chickens. Vet. Microbiol..

[32] Meniai I (2021). Putative antigenic proteins identified by comparative and subtractive reverse vaccinology in necrotic enteritis-causing *Clostridium perfringens* isolated from broiler chickens. BMC Genom..

[33] Kheravii SK, Swick RA, Choct M, Wu SB (2018). Effect of oat hulls as a free choice feeding on broiler performance, short chain fatty acids and microflora under a mild necrotic enteritis challenge. Anim. Nutr..

[34] Díaz Carrasco JM (2016). Use of plant extracts as an effective manner to control *Clostridium perfringens* induced necrotic enteritis in poultry. Biomed. Res. Int..

[35] Imam S, Chen Z, Roos DS, Pohlschröder M (2011). Identification of surprisingly diverse type IV pili, across a broad range of gram-positive bacteria. PLoS ONE.

[36] Varga JJ (2006). Type IV pili-dependent gliding motility in the Gram-positive pathogen *Clostridium perfringens* and other Clostridia. Mol. Microbiol..

[37] Lepp D (2013). Identification of accessory genome regions in poultry *Clostridium perfringens* isolates carrying the netB plasmid. J. Bacteriol..

[38] Craig L, Li J (2008). Type IV pili: paradoxes in form and function. COSB.

[39] Georgiadou M, Castagnini M, Karimova G, Ladant D, Pelicic V (2012). Large-scale study of the interactions between proteins involved in type IV pilus biology in *Neisseria meningitidis*: Characterization of a subcomplex involved in pilus assembly. Mol. Microbiol..

[40] Koch C, Jensen S, Øster A, Houen G (1996). A comparison of the immunogenicity of the native and denatured forms of a protein. APMIS.

[41] Holm BE (2015). Antibodies with specificity for native and denatured forms of ovalbumin differ in reactivity between enzyme-linked immunosorbent assays. APMIS.

[42] Mandlik A, Swierczynski A, Das A, Ton-That H (2008). Pili in Gram-positive bacteria: Assembly, involvement in colonization and biofilm development. Trends Microbiol..

[43] Melville S, Craig L (2013). Type IV pili in Gram-positive bacteria. MMBR.

[44] LeGall J (1988). Isolation and characterization of rubrerythrin, a non-heme iron protein from *Desulfovibrio vulgaris* that contains rubredoxin centers and a hemerythrin-like binuclear iron cluster. Biochem. J..

[45] Morvan C, Folgosa F, Kint N, Teixeira M, Martin-Verstraete I (2021). Responses of *Clostridia* to oxygen: From detoxification to adaptive strategies. Environ. Microbiol..

[46] Rahman N, Islam MM, Kibria MG, Unzai S, Kuroda Y (2020). A systematic mutational analysis identifies a 5-residue proline tag that enhances the in vivo immunogenicity of a non-immunogenic model protein. FEBS Open Biol..

[47] Ledford DK (1994). Indoor allergens. J. Allergy Clin. Immunol..

[48] Dintzis R, Middleton M, Dintzis H (1983). Studies on the immunogenicity and tolerogenicity of T-independent antigens. J. Immunol..

[49] Xu Z-L (2009). Application of computer-assisted molecular modeling for immunoassay of low molecular weight food contaminants: A review. Anal. Chim. Acta..

[50] Spinks C, Wyatt G, Lee H, Morgan M (1999). Molecular modeling of hapten structure and relevance to broad specificity immunoassay of sulfonamide antibiotics. Bioconjug. Chem..

[51] Cooper KK, Songer JG (2009). Necrotic enteritis in chickens: A paradigm of enteric infection by *Clostridium perfringens* type A. Anaerobe.

[52] Pillai-Kastoori L (2020). Antibody validation for Western blot: By the user, for the user. JBC.

[53] Kulkarni R, Parreira V, Jiang Y-F, Prescott J (2010). A live oral recombinant *Salmonella enterica* serovar Typhimurium vaccine expressing *Clostridium perfringens* antigens confers protection against necrotic enteritis in broiler chickens. CVI.

[54] Mot D (2013). Day-of-hatch vaccination is not protective against necrotic enteritis in broiler chickens. Avian Pathol..

[55] Cooper K, Trinh H, Songer JG (2009). Immunization with recombinant alpha toxin partially protects broiler chicks against experimental challenge with *Clostridium perfringens*. Vet. Microbiol..

[56] Kulkarni R, Parreira V, Sharif S, Prescott J (2006). *Clostridium perfringens* antigens recognized by broiler chickens immune to necrotic enteritis. CVI.

[57] Kilkenny C, Browne WJ, Cuthill IC, Emerson M, Altman DG (2010). The ARRIVE guidelines animal research: Reporting in vivo experiments. PLoS Biol..

[58] Team, R.C. *R: A Language and Environment for Statistical Computing*. (R Foundation for Statistical Computing, 2013). https://www.r-project.org/.

