# Supplementary material for: Immunization of broiler chickens with five newly identified surface-exposed proteins unique to Clostridium perfringens causing necrotic enteritis
Source: Sci Rep. 2023 Mar 31;13:5254. doi: 10.1038/s41598-023-32541-4 (PMC10063949; doi:10.1038/s41598-023-32541-4)
Supplement: Supplementary file 1 — Supplementary Information. [file 41598_2023_32541_MOESM1_ESM.pdf]

a

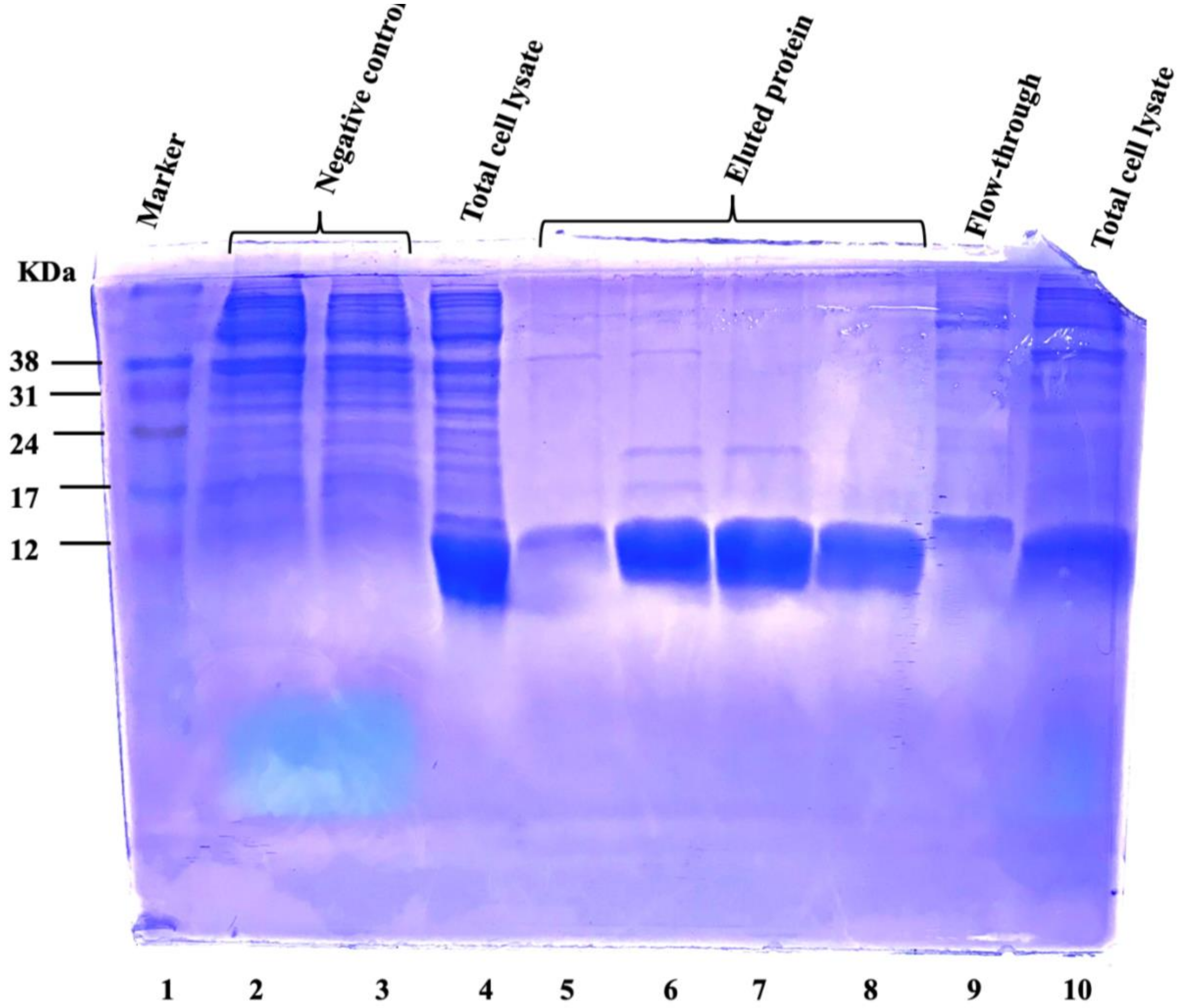

Marker
Negative control
Total cell lysate
Eluted protein
Flow-through
Total cell lysate
KDa
38
31
24
17
12
1
2
3
4
5
6
7
8
9
10

b

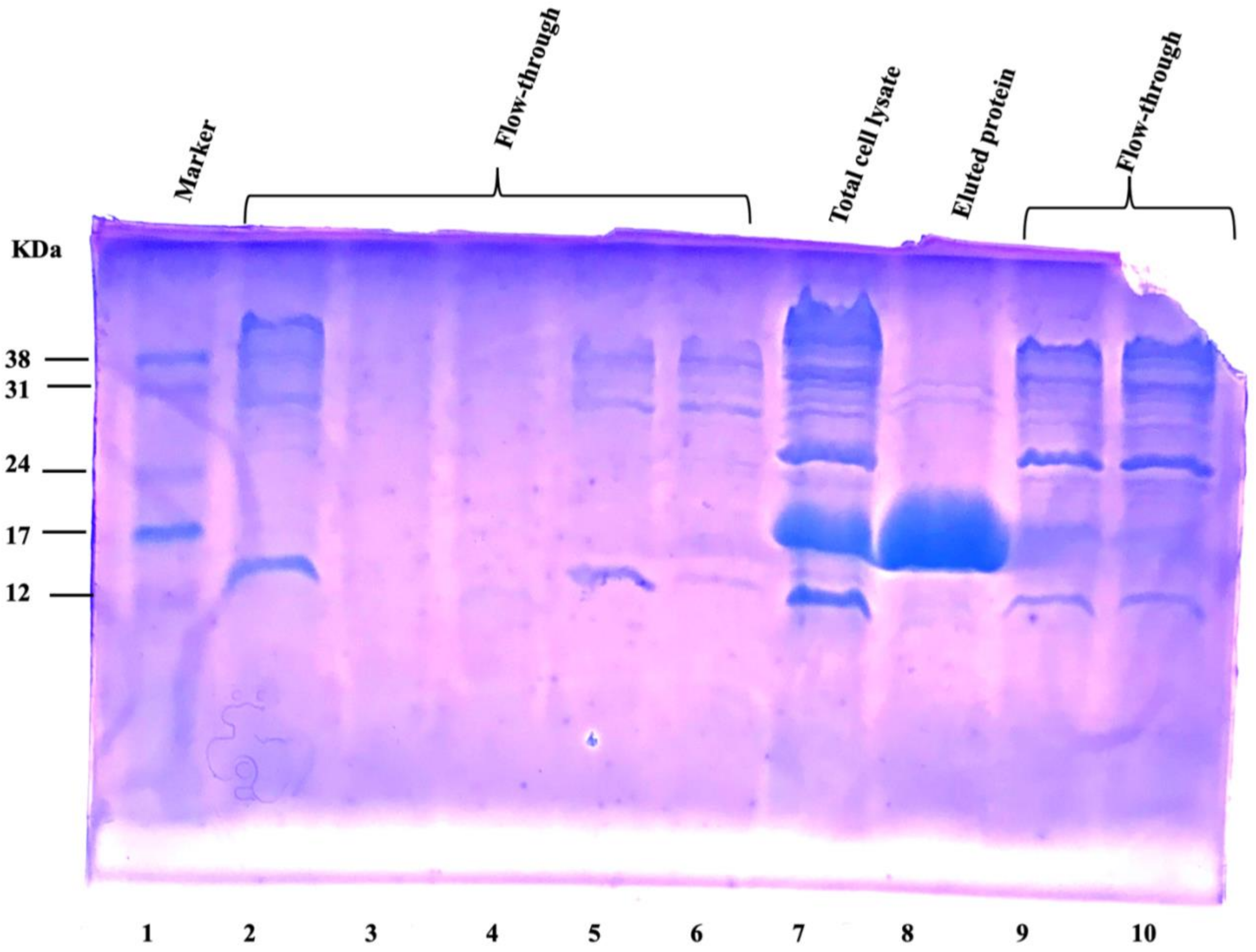
Marker
Flow-through
Total cell lysate
Eluted protein
Flow-through
KDa
38
31
24
17
12
1
2
3
4
5
6
7
8
9
10

c

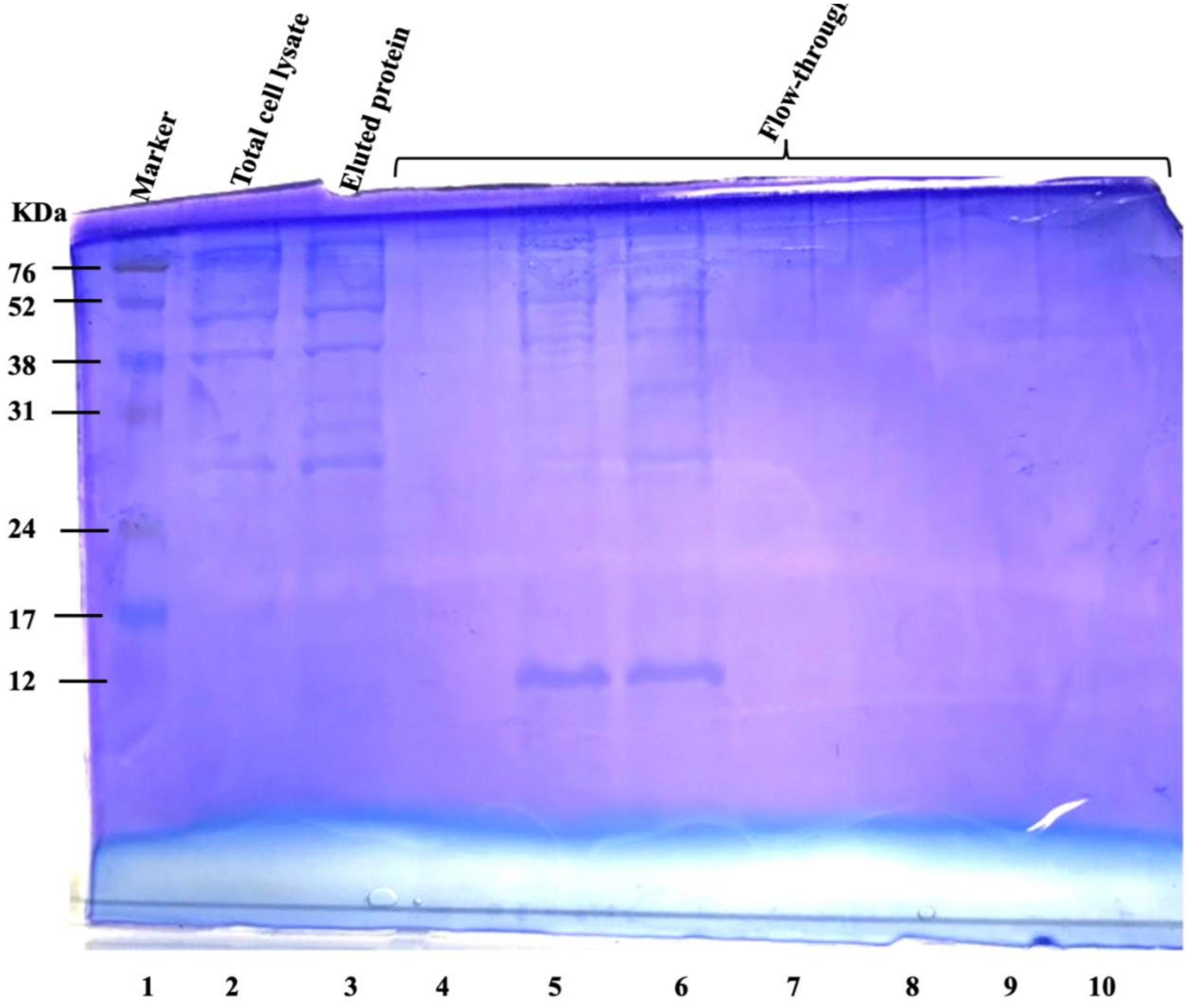
Marker
Total cell lysate
Eluted protein
Flow-through
KDa
76
52
38
31
24
17
12
1
2
3
4
5
6
7
8
9
10

d

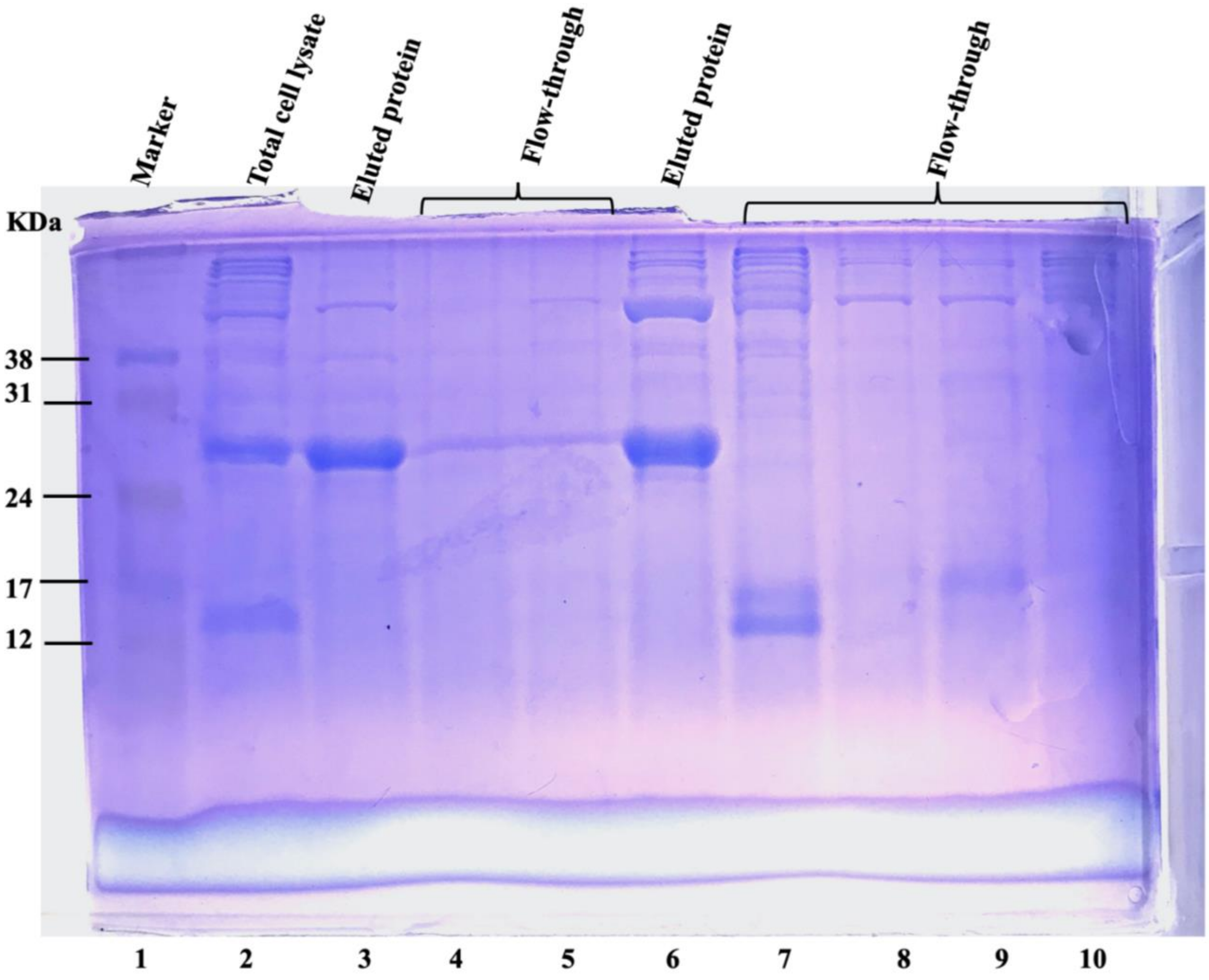
Marker
Total cell lysate
Eluted protein
Flow-through
Eluted protein
Flow-through
KDa
38
31
24
17
12
1
2
3
4
5
6
7
8
9
10

e

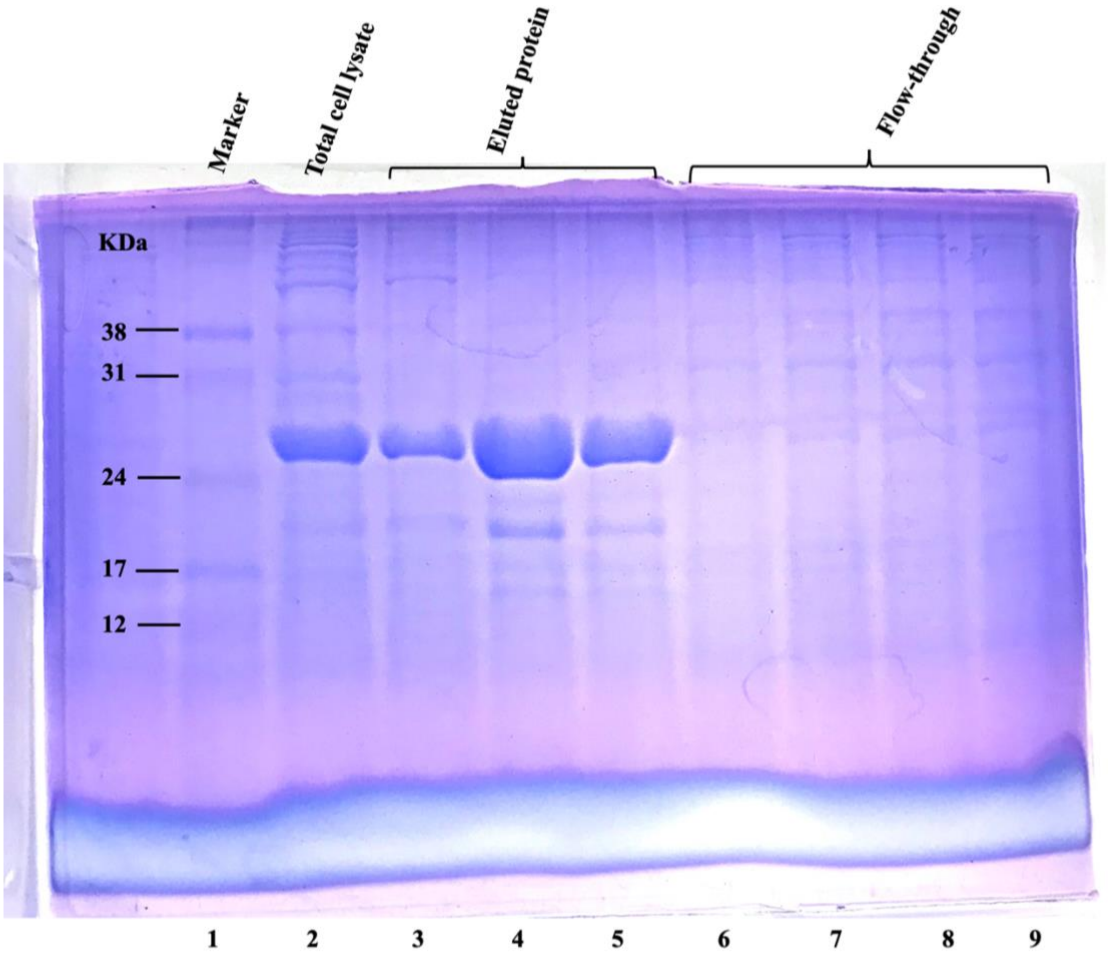

Figure S.1. SDS-PAGE gel showing purification of His-tagged recombinant proteins by imidazole step gradient. 15 µL of total cell lysate and fractions of a) P153, b) P264, c) P509, d) P537, and e) P561 were observed by SDS-PAGE and Coomassie Blue staining. The size of the recombinant protein is 4 KDa higher than the expected size due to the inclusion of the His-tag and V5 tag at N-terminal. Negative control samples in **Fig 1.a** consist of total cell protein samples from BL21 cells that were transformed with pET empty vector.

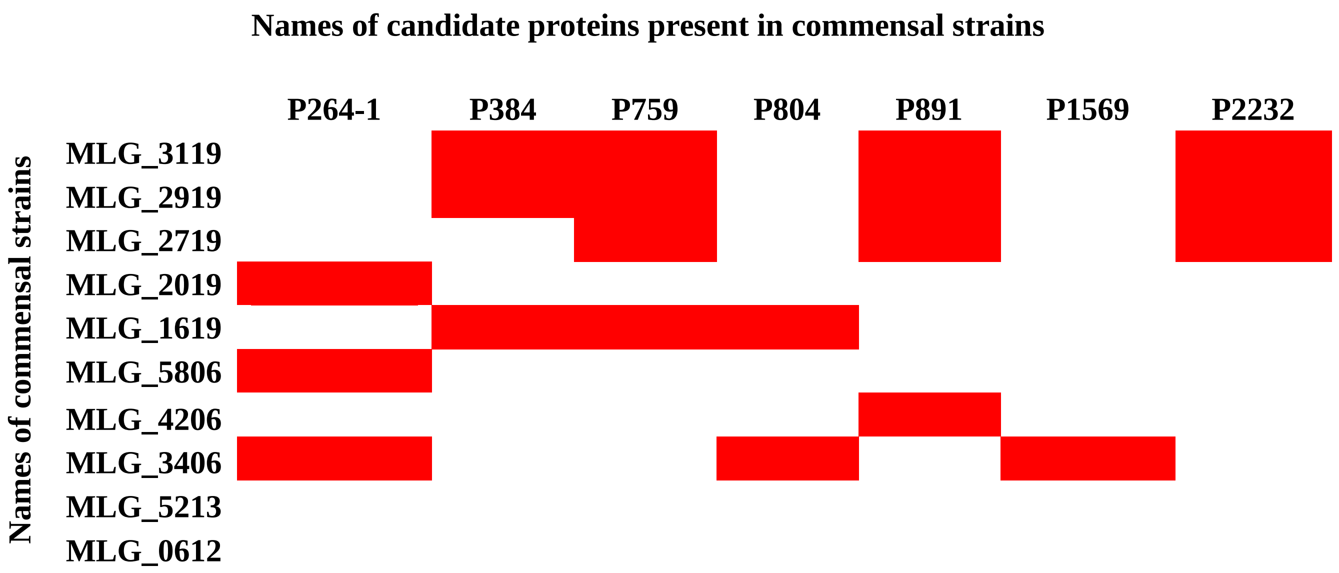

Table S.1 Distribution of candidate protein-encoding genes among analyzed commensal strains of *C. perfringens*.

Name of candidate	Similarity (%) - DNA sequence	Similarity (%) - Protein sequence
P264-1	99%	98%
P384	99%	98%
P759	99%	100%
P804	99%	100%
P891	98%	98%
P1569	97%	99%
P2232	99%	99%
P153	Absent from commensal *C. perfringens* strains analyzed	
P264-2	Absent from commensal *C. perfringens* strains analyzed	
P537	Absent from commensal *C. perfringens* strains analyzed	
P509	Absent from commensal *C. perfringens* strains analyzed	
P561	Absent from commensal *C. perfringens* strains analyzed	
P1074	Absent from commensal *C. perfringens* strains analyzed	
P2091	Absent from commensal *C. perfringens* strains analyzed	

Table S.2. Name of candidate protein-encoding genes and the DNA and protein similarity percentage between *in silico* identified protein-encoding genes and amplicons derived from commensal *C. perfringens* strains.
